# Impact of COVID-19 on Meaning Making of Dementia Caregivers in Hong Kong: From the Generational Perspectives

**DOI:** 10.1093/geroni/igab046.943

**Published:** 2021-12-17

**Authors:** W Q Lou Vivian, Reynold Leung

**Affiliations:** 1 The University of Hong Kong, Hong Kong, Hong Kong; 2 The University of Hong Kong, Pokfulam, Hong Kong

## Abstract

This study examined the impact of COVID-19 on meaning making among adult children dementia caregivers and the association with caregiver mental well-being. Adult caregivers (n=601) from two generations, 1946-1964 (Baby Boomers) and 1965-1980 (Generation X), were recruited in Hong Kong between October 2019 and June 2020. Participants were assessed on depressive symptoms (PHQ-9) and meaning making (Finding Meaning Through Caregiving Scale-FMTC). Generation X scored higher on sense of loss (p = 0.04) and lower on provisional meaning of FMTC (p=0.017). Moreover, an interaction effect (p=0.003) between generation and COVID-19 were found. During the pandemic, Generation X caregivers were more likely to suffer from higher losses, higher depressive symptoms (>23.2% moderate to severe) and lower provisional meaning (p=0.03) compared to their boomer counterparts. The level of meaning making is more important to Generation X caregivers, especially in COVID-19 situation. Government should consider generation-responsive services and education support in guiding service implementation.

